# Effects of Mindfulness-Based Therapy on Clinical Symptoms and DNA Methylation in Patients with Polycystic Ovary Syndrome and High Metabolic Risk

**DOI:** 10.3390/cimb45040178

**Published:** 2023-03-24

**Authors:** Hana Dema, Alja Videtič Paska, Katarina Kouter, Mojca Katrašnik, Mojca Jensterle, Andrej Janež, Aleš Oblak, Borut Škodlar, Jurij Bon

**Affiliations:** 1 Department of Health Care Quality, University Medical Centre Ljubljana, SI-1000 Ljubljana, Slovenia; 2 Institute for Biochemistry and Molecular Genetics, Faculty of Medicine, University of Ljubljana, SI-1000 Ljubljana, Slovenia; 3 Department of Endocrinology, Diabetes and Metabolic Diseases, University Medical Centre Ljubljana, SI-1000 Ljubljana, Slovenia; 4 Department of Internal Medicine, Faculty of Medicine, University of Ljubljana, SI-1000 Ljubljana, Slovenia; 5 University Psychiatric Clinic Ljubljana, SI-1260 Ljubljana, Slovenia; 6 Department of Psychiatry, Faculty of Medicine, University of Ljubljana, SI-1000 Ljubljana, Slovenia

**Keywords:** mindfulness-based stress reduction program, polycystic ovary syndrome, epigenetics, DNA methylation, candidate genes, depression, anxiety

## Abstract

Polycystic ovary syndrome (PCOS) is an endocrine and metabolic disorder affecting women of reproductive age. Research has shown that epigenetic alterations such as DNA methylation may play a role in the development and progression of abnormal ovarian function and metabolic disorders in PCOS. Studies have identified specific genes (related with insulin signaling and steroid hormone metabolism) that are methylated in women with PCOS. DNA methylation appears to respond to various interventions aimed at altering health and lifestyle factors. We tested the efficacy of a mindfulness-based stress reduction program (MBSR) in PCOS patients. We examined its effects on anthropometric measurements, mental health and wellbeing, and alterations in DNA methylation in peripheral blood. MBSR was associated with a reduction in body mass index, waist circumference and blood glucose level, an improvement in subjectively perceived general health, emotional role limitation, and levels of pain, as well as mindfulness-like traits. MBSR reduced the expression of anxious symptomatology and subjectively perceived stress. Methylation changes were observed in four genes: COMT, FST, FKBP51, and MAOA. We conclude that MBSR may be a useful supplementary therapy to mitigate the deleterious effects of PCOS on mental health.

## 1. Introduction

Polycystic ovary syndrome (PCOS) is a common endocrine and metabolic disorder that occurs in 5–18% of women of reproductive age [1,2]. It is characterized by the presence of heterogeneous reproductive, metabolic, and androgenizing symptoms that may manifest in complex phenotypes. The most common clinical symptoms are obesity, androgenization, menstrual cycle disorders, and infertility [3]. Various signs of metabolic syndrome may be present [4]. The expression of symptoms is strongly influenced by obesity, which is present in 40–80% of PCOS patients [5]. Obesity increases insulin resistance (IR) [6], which, together with impaired insulin sensitivity and beta cell function, leads to accelerated development of prediabetes and type 2 diabetes in 30–45% of patients [7]. In the presence of metabolic syndrome, patients are also at risk of developing cardiovascular disease, non-alcoholic fatty liver disease, endometrial hyperplasia and cancer, as well as some other cancers [7,8]. Comorbid mental disorders are also common, especially depression, which occurs in 40% of patients [9]. The development of depression and anxiety disorders is significantly influenced by patients’ low self-esteem due to physical symptoms, which cause problems in social life and sexuality [10]. The exact pathogenesis of depression in PCOS may be more complex and dependent on other pathological mechanisms of the disorder [11].

The etiologic mechanisms of PCOS are not clear. In addition to hypothalamic and ovarian dysfunction [2,11], androgenization [12], dysfunction of adipose tissue [13], insulin resistance in skeletal muscle [14], elevated serum levels of inflammatory markers [15], and changes in the endometrium are present [16]. The cause of PCOS is complex and includes genetic and epigenetic susceptibility. Several studies have shown the influence of genetic and environmental factors, starting early in life [1,17]. Epigenetic alterations such as DNA methylation, histone modifications, and changes in noncoding RNA activity have been detected in various tissues of patients with PCOS [18]. DNA methylation appears to play an important role in the pathogenesis of PCOS. Studies have reported alterations in DNA methylation in ovarian [18] and adipose tissue [19], skeletal muscle [20], as well as in peripheral blood [21] and umbilical cord blood [22]. These DNA methylation changes likely participate in the deregulation of genes involved in inflammation, hormone synthesis and signaling, and glucose and lipid metabolism [18].

Epigenetic changes can be altered by various environmental factors such as physical activity, stress, or medical interventions such as medication, psychotherapy, or various meditation-based interventions [23,24]. Although epigenetic marks are considered relatively stable, their changes can be very dynamic and respond rapidly to stress, diet, exercise, or lifestyle-modifying therapeutic interventions [25]. In PCOS, recent studies in animal models and patient populations have shown that therapeutic interventions can influence DNA methylation and phenotypic changes in tissues affected by the disease [26,27], raising the possibility of therapeutic interventions Recently, there has been a resurgence of interest in traditional mind–body therapeutic practices as psychological interventions for various chronic medical conditions [28]. Mind–body interventions (MBI) are a group of diverse techniques that focus on the interactions between mind, brain, body, and behavior and aim to reduce stress and increase acceptance through long-term personality changes [29]. Some of them include an active physical component [30], while others focus mainly on meditation and relaxation techniques [31,32]. The majority of MBIs for medical disorders appear to be based on mindfulness-related approaches. Mindfulness can refer to both a dispositional trait and a meditation practice that cultivates awareness of the present moment [33,34]. Health interventions based on mindfulness can include formal programs such as Mindfulness-Based Stress Reduction (MBSR) and Mindfulness-Based Cognitive Therapy [35], or be part of other therapeutic techniques such as Acceptance and Commitment Therapy [36]. Like other MBIs, they can reduce stress, regulate emotional processes in depression and anxiety, increase motivation, and influence cognitive processes to promote adaptations and compliance in chronic disease management [37,38]. Mindfulness, when practiced over time, promotes trait-related changes in attention, awareness, perception, appraisal, and self-regulation [39]. At the cellular level, significant epigenetic changes in DNA methylation patterns may occur in genes related to the immune system, inflammation, and aging [40,41].

Management of weight and lifestyle factors such as diet, physical activity, and psychological well-being are first-line therapies in evidence-based guidelines for PCOS [42]. Mindfulness-based stress management programs have been proposed and successfully used in women with PCOS to reduce their levels of stress, anxiety, and depression [43,44] and to improve their self-efficacy related to diet and physical activity [45]. To our knowledge, no study to date has attempted to examine the effects of mindfulness on epigenetic changes in PCOS. We conducted a randomized clinical trial to determine the efficacy of a mindfulness-based stress reduction program in PCOS patients and to investigate its effects on changes in DNA methylation in peripheral blood. Candidate genes were selected considering those shown to be affected in PCOS and compared with genes affected in stress-related disorders (see Section 2).

## 2. Materials and Methods

### 2.1. Participants

Forty-two female patients with a mean age of 40.1 years (SD = 7.3) and an average of 14.1 years of education (SD = 1.7) were recruited from the Department of Endocrinology, Diabetes and Metabolic Diseases at University Medical Centre Ljubljana and randomly assigned to the experimental group participating in MBSR program (N = 21) or to the control group on the waiting list (N = 21) (see Table 1 for descriptive statistics on demographic, clinical and laboratory characteristics of participants at baseline). All patients were diagnosed with PCOS according to the ASRM-ESHRE Rotterdam Consensus Criteria (Rotterdam ESHRE/ASRM-Sponsored PCOS Consensus Workshop Group, 2004). Diagnoses were confirmed by experienced clinicians (MJ and AJ) involved in the study. The following exclusion criteria applied to participants: current moderate or severe depressive episode, neurologic disorder or traumatic brain injury, substance abuse, current metformin therapy, pregnancy, chronic kidney or liver disease. Infertility was present in 10 patients. Metabolic syndrome was present in 28 patients according to the modified NCEP-ATP III criteria [46]. In accordance with the exclusion criteria, none of the participants were formally diagnosed with a psychiatric disorder such as depression or anxiety at the beginning of the study or in the past.

The study was approved by the Medical Ethics Committee and all participants signed an informed consent form (see Institutional Review Board Statement).

### 2.2. Study Design

**Figure 1:**
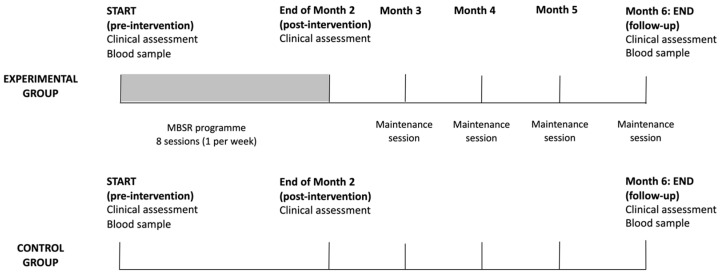
Study design.

We conducted a clinical assessment of all patients at three time points in the study—at baseline (pre-intervention), at the end of the MBSR program at 2 months (post-intervention), and at the end of the study at 6 months (follow-up) (see Figure 1). Clinical assessment included measurements of height, weight, waist circumference, systolic and diastolic blood pressure (from which we calculated mean arterial blood pressure, MAP), and heart rate. Mental health status was assessed with clinical scales for depression (Beck Depression Inventory-II [47]) and anxiety (Beck Anxiety Inventory [48]), perceived stress (Stress Questionnaire [49]), quality of life (RAND-36 [50,51]), and trait-like tendencies of mindfulness in daily life (Five-Facet Mindfulness Questionnaire [52]). Perceived stress was measured as a cumulative score on a 20-item Stress Questionnaire, with possible scores ranging from 0 to 20 [49].

Blood samples were collected at the beginning and end of the study. We determined serum levels of cholesterol, HDL, LDL, triglycerides, fasting glucose, and postprandial glucose after 120 min.

Participant properties of the experimental and control groups are summarized in Table 1. Control group was, on average, statistically significantly younger, and had a lower level of triglycerides.

### 2.3. Mindfulness-Based Stress Reduction Program

Patients in the experimental group participated in a mindfulness-based stress reduction (MBSR) program (see Figure 1). Therapeutic sessions were held once a week for 8 weeks at the Department of Mental Health at the University Psychiatric Clinic Ljubljana. They were led by experienced therapists who were not otherwise involved in the study. The content of the standard MBSR program at the department consists of 8 group meetings (once a week, two hours) and one individual meeting to clarify dilemmas and open questions and to anchor the practice of mindfulness in everyday life. The structure of the group meeting is as follows: Beginning with formal practice, review of practice and experience at home, discussion and group practice related to the theoretical theme of the weekly meeting and concluding with mindfulness practice. Participants performed specific exercises and techniques for developing mindfulness, such as practicing mindfulness in relation to loving kindness and breathing, in everyday activities and walking, awareness of body sensations (body scan) and simple body positions. To eliminate confounding factors, participants were instructed to maintain their usual diet, medications, and exercise levels during the mindfulness-based treatment.

### 2.4. DNA Methylation Analysis

#### 2.4.1. Selection of Candidate Genes and Target Sequences

DNA methylation analysis was performed for several candidate genes selected for their association with reproduction, obesity, metabolic syndrome, and diabetes or their relevance to stress-related disorders and mood disorders. The genes and their functional significance are listed in Table 2.

Mapping of target sequences was performed using the UCSC Genome Browser, Human Genome Build 19 (GRCh37/hg19) [53]. Target sequences in candidate genes were located in the CpG islands (CGI), with additional 500-base-pair flanking regions upstream and downstream of the CGI. For genes lacking specific CpG islands, the target sequence was located near the transcription start site, where sequences with potential regulatory function are located.

**Table 2:** Amplicon positions of candidate genes and their functional significance.

Amplicon	Position (Human Genome Build 19) and Length of Target Sequence	Number of CpG Islands	Functional Significance
BDNF-81_1	chr11:27744260-27744605 (−), 346	22	Brain-derived neurotrophic factor gene; regulates growth, differentiation, maintenance, death/survival and plasticity of neurons [54]
BDNF-81_2	chr11:27743702-27743960 (−), 259	10	
BDNF-81_3	chr11:27743454-27743762 (−), 309	20	
BDNF-14_1	chr11:27741988-27742250 (−), 263	13	
BDNF-58_1	chr11:27740916-27741131 (−), 216	16	
BDNF-58_2	chr11:27740607-27740901 (−), 295	30	
BDNF-95_1	chr11:27721638-27721854 (−), 217	19	
BDNF-95_2	chr11:27722466-27722696 (−), 231	13	
BDNF-95_5	chr11:27722209-27722487 (−), 279	23	
CEBPB_1	chr20:48807584-48807968 (+), 385	52	Transcription factor gene; participates in the ovarian follicle development and insulin signaling [55]
CEBPB_2	chr20:48807389-48807650 (+), 262	23	
COMT_1	chr22:19951071-19951343, 273	14	Catechol-O-methyl transferase (COMT) gene, determines prefrontal dopaminergic availability [56]
COMT_2	chr22:19929042-19929349, 308	36	
COMT_4	chr22:19950002-19950320, 319	13	
EPHX1_2	chr1:225998005-225998262 (+), 258	25	Epoxide hydrolase-1 gene; regulates steroid synthesis pathways associated with PCOS [57]
EPM2A_1	chr6:146056330-146056621 (−), 292	36	Dual-specificity phosphatase gene; involved in the regulation of glycogen metabolism [55]
FKBP51_1	chr6:35656629-35656978 (−), 350	38	FK506 binding protein 5 gene; regulates glucocorticoid activity and acute stress response [58]
FST_1	chr5:52775484-52775780 (+), 297	23	Follistatin, an activin-binding protein gene; associated with PCOS [59]
FST_2	chr5:52776111-52776302 (+), 192	13	
FST_3	chr5:52776279-52776668 (+), 390	54	
HTR1A_2	chr5:63257662-63257938 (−),277	15	Serotonin receptor 1A gene; regulates serotonin system function [60]
HTR1A_3	chr5:63256777-63257065 (−), 289	20	
IGFBP1_1	chr7:45928046-45928301 (+), 256	21	Insulin-like growth factor binding protein gene; regulates cell migration and metabolism [55]
IGFBP1_2	chr7:45928280-45928533 (+), 254	35	
INSR_1	chr19:7293426-7293764 (−), 339	36	Insulin receptor gene; associated with insulin resistance in PCOS [61]
LHCGR_1	chr2:48982757-48983019 (−), 263	16	Luteinizing hormone/choriogonadotropin receptor gene; involved in human gonadal maturation and function [62]
MAOA_2	chrX:43513981-43514236, 256	18	Monoamine oxidase A gene; involved in serotonin degradation [60]
MAOA_3	chrX:43515510-43515787, 278	13	
NR3C1_1	chr5:142783586-142783906 (−), 321	39	Glucocorticoid receptor gene; associated with regulation of HPA axis and stress response [63]
PPARG1A_1	chr4:23890471-23890804 (−), 334	9	Peroxisome proliferator-activated receptor gamma 1 gene; regulates ovarian function [64]
SLC6A4_3	chr17:28562753-28563050 (−), 298	29	Serotonin transporter gene; associated with depression treatment outcomes [58]
SLC6A4_5	chr17:28563277-28563552 (−), 276	7	
TBKBP1_1	chr17:45772538-45772753 (+), 216	9	*TBKBP1* gene; involved in the *TNF-α*/*NF-κB* pathway activated under conditions of acute and chronic psychological stress [40]
TPH2_1	chr12:72332514-72332805 (+), 292	9	Tryptophan hydroxylase 2 gene; serotonin synthesis rate limiting enzyme [60]

#### 2.4.2. DNA Isolation and Bisulfite Conversion

Venous blood tubes were labeled with a code to ensure confidentiality of the data. They were stored at −70 °C until DNA isolation. DNA was isolated from 100 μL whole blood using the QIAcube robotic workstation and the commercially available QIAmp DNA Mini Kit (Qiagen, Venlo, The Netherlands) according to the supplier’s instructions and eluted in 100 μL of ultra clean water. A total of 1000 ng of DNA was bisulfite converted using the EpiTect Fast Bisulfite Kit (Qiagen, Venlo, The Netherlands) and eluted in 50 μL of elution buffer, yielding DNA at a final concentration of 20 ng/μL.

#### 2.4.3. Primer Design

Primers were designed using Methyl Primer Express v1.0 [65], which allows the design of primers for bisulfite-converted DNA. Each primer pair was designed to amplify regions approximately 200–350 basepairs long. If the CGI were greater than 300 bp, two or more primer pairs were designed to cover the CGI sequence as much as possible. Table 2 shows the amplicon positions, amplicon lengths and the number of CpGs.

Primer properties and specificity were determined using BiSearch [66] and the IDT Oligo Analyzer [67]. In the final step of primer design, the Illumina adapter overhang sequences were added to the 5′ end of the DNA sequence-specific primer and the final primer sequences were rechecked for their properties and specificity.

#### 2.4.4. Amplicon Generation and Sequencing

Amplicon library preparation followed Illumina’s 16S protocol with some modifications [68]. The target sequences were amplified in two rounds of PCR. The first round of PCR was used to generate target sequences (complete primer pair sequences and annealing temperatures in Supplementary Table S1). The total volume of PCR reactions was 25 μL and was composed of 12.5 μL KAPA HiFi HotStart Uracil+ ReadyMix (Roche, KAPA Biosystems Ltd., Cape Town, South Africa), 1 μM primer, and 20 ng DNA. The PCR protocol was performed as follows: Activation for 5 min at 95 °C, followed by 35 amplification cycles (denaturation for 30 s at 98 °C, annealing for 15 s at a temperature dependent on the primer pair and extension for 15 s at 72 °C), and concluded by a final extension for 1 min at 72 °C and holding at 4 °C. Annealing temperatures for each specific primer pair are listed in Supplementary Table S1. Amplicons from the first round of PCR were visualized on 2% agarose gel electrophoresis to confirm amplification of fragments of the correct length. Shorter, nonspecific amplification fragments were removed using AMPure XP beads (Beckman Coulter, Brea, CA, USA). Purified amplicons from each subject were pooled in a tube to form an equimolar pool (concentrations were measured using Quant-iT PicoGreen dsDNA (Thermo Scientific, Life Technologies, Waltham, MA, USA)).

The second round of PCR was used to label each subject with a specific identifying sequence that would allow multiplexing during the sequencing run. Nextera XT v2 Index Set A and Set D primers (Illumina, San Diego, CA, USA) were used to label the pooled samples. The total volume of PCR reactions was 50 μL and was composed of 25 μL KAPA HiFi HotStart Uracil+ ReadyMix (Roche, Basel, Switzerland), Nextera XT v2 primers, and 4 ng of equimolar amplicon pool. The PCR protocol was performed: Activation for 45 s at 98 °C, 10 amplification cycles (denaturation for 15 s at 98 °C, annealing for 30 s at 55 °C, and extension for 30 s at 72 °C), followed by a final extension for 1 min at 72 °C. Generation of amplicons of appropriate length was again confirmed by 2% agarose gel electrophoresis.

#### 2.4.5. Library Preparation and Sequencing

Amplicons from the second round of PCR amplification were subjected to size selection (AMPure XP paramagnetic beads, Beckman Coulter, Brea, CA, USA), followed by concentration determination. The concentration of each subject library was measured using an ultrasensitive fluorescent nucleic acid dye that allowed quantification of double-stranded DNA (PicoGreen dsDNA quantification reagent, Thermo Fisher, Waltham, MA, USA). The final library was prepared by equimolar pooling of the individual subject libraries to a final library with a molar concentration of 10 nM. The final library was diluted and denatured according to the recommendations in the Illumina MiniSeq System Denature and Dilute Libraries Guide. The final library was sequenced on the Illumina MiniSeq sequencer using the MiniSeq Mid Output Kit (300 cycles) with 150 bp paired-end reads.

### 2.5. Bioinformatic and Statistical Analysis

Raw sequencing reads (in FASTQ format) were quality checked using the FastQC tool (v0.14.1) [69]. Trim galore (v0.6.7) [70] was used to trim bases of insufficient quality (Q score below 30) and adapter sequences. Bismark (v0.21.0) [71] was used to align these trimmed sequences to the reference genome UCSC Genome Browser (Homo sapiens version hg19).

Aligned reads were further analyzed using the R environment (v4.0.4) [72], the methylKit package [73], and the methylSig package [74], with data corrected for age and multiple testing. Differentially methylated CpGs (DMC) were identified by comparing the percent DNA methylation for each CpG cytosine between our two assay groups. The average DNA methylation values of the amplicon were calculated using the values of all CpGs in that amplicon and compared between the two groups of subjects studied. The normality of the distribution of CpG values was tested using the Shapiro–Wilk normality test. Because the distribution was nonparametric for most of the amplicon data, differences in the percentages of mean methylation of the amplicons between groups were calculated using the Mann–Whitney U test and the Benjamini–Hochberg correction. Corrected *p* values ofless than 0.05 were considered statistically significant.

Statistical analysis was performed using custom scripts written in R (version x64 4.1.2) [72] and RStudio (version 4.1.2) [75]. A between-group and within-subjects design was used. Accordingly, data were analyzed with mixed ANOVA. Data were first tested for assumption violation. The identify outlier function from the R package rstatix was used to automatically detect outliers. Shapiro–Wilk test was used to test for normality, followed by a visual inspection of the QQ plot. Levene’s test was used to test for homogeneity of variance. Box’s M test was used to test homogeneity of covariance. Mauchly’s test was used to test sphericity. When parametric assumptions were met, a two-way mixed method ANOVA was used to test for differences between groups and within subjects. When parametric assumptions were not met, the robust mixed ANOVA implemented in the R package WRS2 [76] was used.

When a significant interaction was detected, post hoc tests were performed. Bonferroni correction for multiple comparisons was applied to each test. The effect of group at each time point and the effect of time at each level of the grouping variable were estimated using one-way ANOVA. Pairwise comparisons between grouping levels were performed using a pairwise T test. Finally, a pairwise *t* test was used for pairwise comparisons between time points at each level of the grouping variable.

## 3. Results

### 3.1. Effects of the MBSR Program on Physical Health and Quality of Life

**Figure 2:**
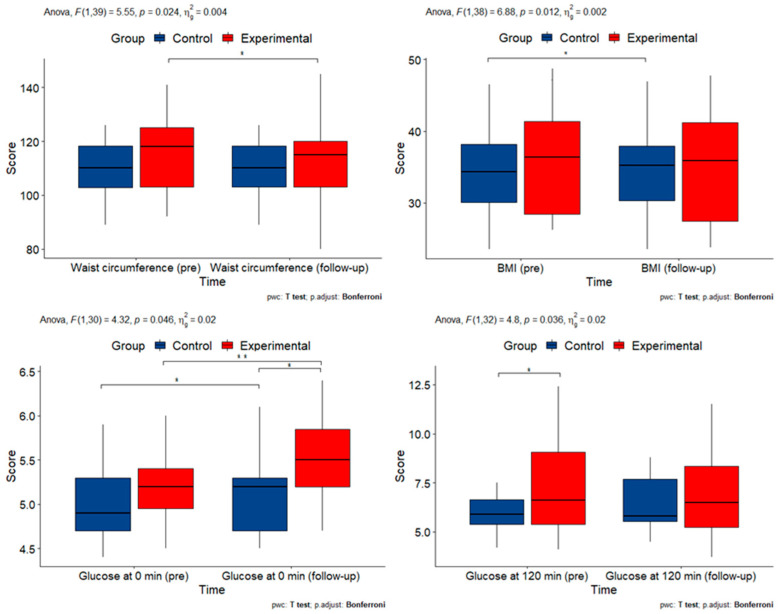
Box plots of clinical and laboratory measurements with statistically significant interactions (BMI: body mass index; min: minutes; pre: pre-intervention). Asterisks denote statistically significant pairwise comparisons.

Eleven clinical and laboratory measures were obtained. A two-way mixed ANOVA was performed to evaluate the effects of time and group on each measurement. The results are summarized in Table 3 and Figure 2.

A two-way mixed ANOVA was performed to evaluate the effects of time and group on all clinical and laboratory measurements except triglycerides, for which a robust mixed ANOVA was used because the data were nonparametric. For weight (F(1, 40) = 5.032, *p* = 0.030), BMI (F(1, 39) = 7.196, *p* = 0.011), waist circumference (F(1, 39) = 5.546, *p* = 0.024), P-glucose at 0 min (F(1, 30) = 4.316, *p* = 0.046) and P-glucose at 120 min (F(1, 33) = 4.643, *p* = 0.039) were statistically significant. This indicates that the intervention affected these measures, but not MAP, heart rate, cholesterol, S-HDL, and S-LDL. For triglycerides, we can assume an effect of the intervention because the groups were significantly different at baseline and did not differ after the intervention.

Quality of life was assessed using the SF-36 questionnaire (RAND-36 version), which consists of eight subscales: physical functioning, physical role limitations, emotional role limitations, energy/fatigue, emotional well-being, social functioning, pain, and general health. There was a statistically significant interaction between time and group on measures of emotional role limitations (F(1.04, 32.39) = 12.85, *p* < 0.001), pain (F(1.14, 42.27) = 4.923, *p* = 0.034), and general health (F(1.56, 62.6) = 6.581, *p* = 0.005), but not physical well-being (F(1, 66) = 48.09, *p* = 0.509) or robust estimation of physical limitations (*p* = 0.593), energy/fatigue (F(1.31, 52.22) = 3.106, *p* = 0.073), and emotional well-being (F(1.39, 55.43) = 2.525, *p* = 0.107). To determine whether the intervention had a longitudinal effect, pairwise comparisons were performed between time points at each group level after a Bonferroni correction for multiple comparisons. Scores for emotional limitations differed significantly between the beginning of the study (pre) and after the intervention (post) (*p* = 0.014) and between the beginning of the study and at follow-up (*p* = 0.014) between the experimental and control groups. Pain scores differed significantly between the experimental and control groups between the beginning of the study and after the intervention (*p* = 0.033) and between the beginning of the study and at follow-up (*p* = 0.03). General health scores differed significantly between the experimental and control groups between the beginning of the study and after the intervention (*p* = 0.011) and between the end of the intervention and at follow-up (*p* = 0.002). This suggests that there was a longitudinal effect of the intervention on pain, general health status, and emotional limitations.

**Figure 3:**
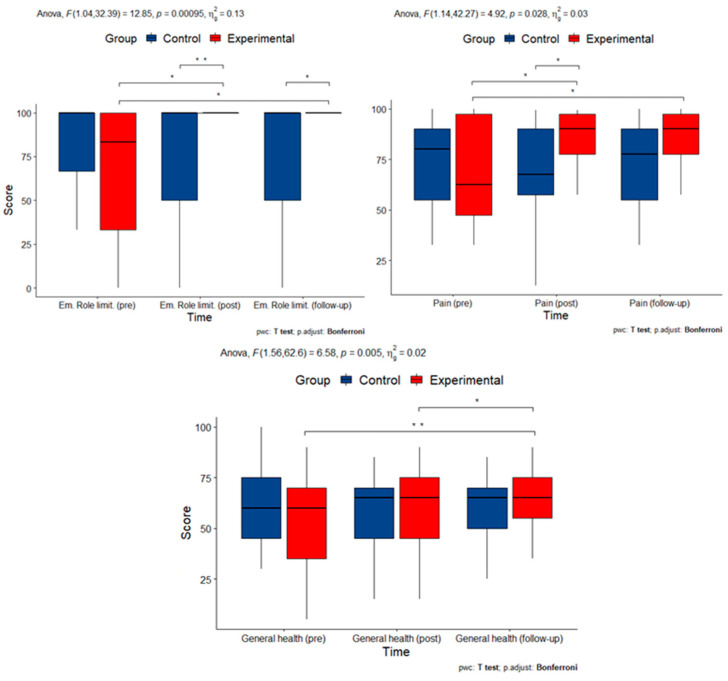
Box plots of measurements of quality-of-life indices with statistically significant interactions (Em. Role: Emotional Role subscale; pre: pre-intervention; post: post-intervention). Asterisks denote statistically significant pairwise comparisons.

Measurements with statistically significant interactions are summarized in Figure 3.

### 3.2. Effects and Interactions of the MBSR Program on Psychological Well-Being

The severity of anxious symptomatology was assessed with BAI. There was a statistically significant two-way interaction between time and group (F(1, 39) = 5.525, *p* = 0.016). Considering the Bonferroni-adjusted *p*-value and using pairwise paired *t*-test comparisons, it can be seen that for the experimental group, the mean BAI score was statistically significantly different between the beginning of the study and after the intervention (*p* = 0.009) and between the beginning of the study and at follow-up (*p* = 0.022). This indicates an immediate reduction in anxiety symptomatology that remained stable over time.

Depressive symptomatology was measured by BDI-II. Scores from BDI-II were nonparametric. To assess the effects of time and group on the scores of BDI-II, the robust estimation mixed ANOVA was used. There were no statistically significant interactions between time and group (*p* = 0.929) or main effects of group (*p* = 0.163) or time (*p* = 0.492).

Finally, there was a statistically significant two-way interaction between time and group (F(2, 72) = 6.911, *p* = 0.002) on scores of perceived stress. Considering the Bonferroni adjusted *p*-value and using pairwise paired *t*-test comparisons, we note that for the experimental group, the mean stress score was statistically significantly different between the beginning of the study and after the intervention (*p* = 0.003) and between the beginning of the study and at follow-up (*p* = 0.000).

**Figure 4:**
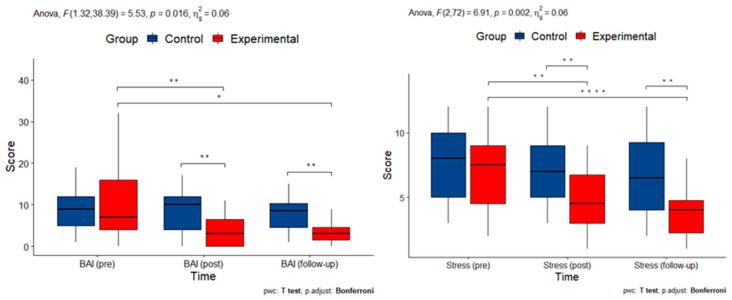
Box plots of measurements of mental health indices with statistically significant interactions (BAI: Beck Anxiety Inventory; Stress: Perceived Stress score; pre: pre-intervention; post: post-intervention). Asterisks denote statistically significant pairwise comparisons.

Measurements with statistically significant interactions are summarized in Figure 4.

### 3.3. Effects and Interactions of the MBSR Program on Mindfulness-Like Traits

Mindfulness-like traits were assessed with the FFMQ questionnaire, which includes five subscales: Observing, Describing, Acting, Non-judging, and Non-reactivity. There was a statistically significant interaction between time and group on measures of Observing (F(1.43, 53.98) = 13.495, *p* < 0.001), Describing (F(2, 80) = 7.563, *p* = 0.018), Acting (F(2, 80) = 4.217, *p* = 0.026), and Non-reactivity (F(1.63, 47.31) = 14.976, *p* < 0.001), but not on Non-judging (F(1.65, 62.85) = 1.088, *p* = 0.333). To determine whether the intervention had a longitudinal effect, pairwise comparisons were performed between time points at each group level after a Bonferroni correction for multiple comparisons. Observing scores were statistically significantly different between the beginning of the study and after the intervention (*p* < 0.001) and between the beginning of the study and at follow-up (*p* < 0.001) in the experimental group. The scores for Describing were statistically significantly different between the beginning of the study and after the intervention (*p* < 0.001) and between the beginning of the study and at follow-up (*p* = 0.008) in the experimental group. The scores for Acting were statistically significantly different between the beginning of the study and after the intervention (*p* = 0.016) and between the beginning of the study and at follow-up (*p* = 0.001) in the experimental group. Non-reactivity scores differed statistically significantly between the beginning of the study and after the intervention for the experimental group (*p* < 0.001) and between the beginning of the study and at follow-up (*p* < 0.001). These results suggest that mindfulness-like traits of observing, describing, acting, and non-reactivity were improved by the intervention and remained stable over time.

**Figure 5:**
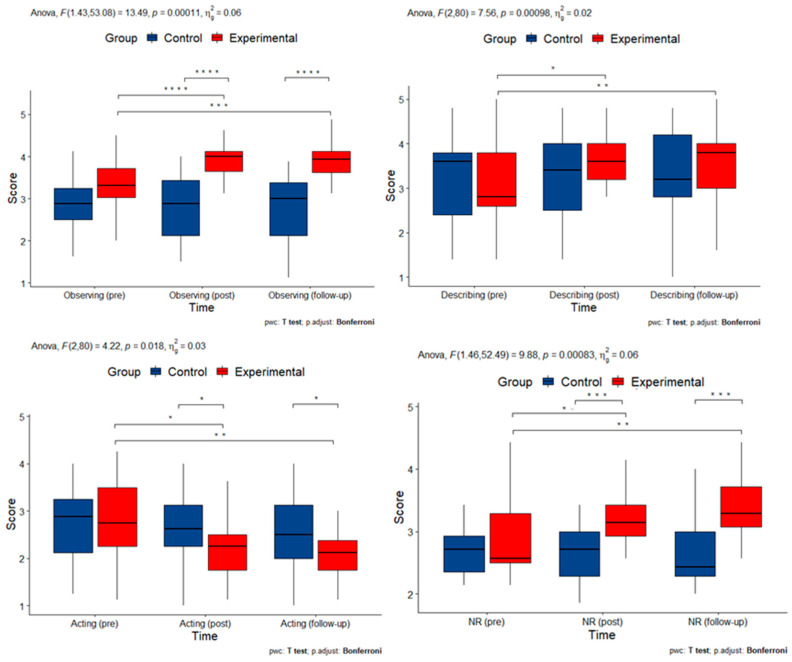
Box plots of measurements of mindfulness-like traits with statistically significant interactions (pre: pre-intervention; post: post-intervention; NR: Non-reactivity subscale). Asterisks denote statistically significant pairwise comparisons.

Measurements with statistically significant interactions are summarized in Figure 5.

### 3.4. DNA Methylation Alterations in Candidate Genes

Mixed ANOVA or robust mixed ANOVA estimation (in the case of nonparametric data) were used to test for differences in the level of DNA methylation of candidate gene amplicons six months after the start of the therapeutic intervention. Table 4 summarizes the *p*-values for the significance of interactions of group and time for candidate gene methylation. Several of the original amplicons could not be included in the analysis due to missing methylation data. A mixed two-way ANOVA was performed to evaluate the effects of time and group on methylation levels. There was a statistically significant two-way interaction between time and group on the methylation of several gene amplicons: COMT_4 (F(1, 28) = 5.667, *p* = 0.075), FST_1 (F(1, 15) = 4.597, *p* = 0.049), FKBP51_1 (F(1, 23) = 4.569, *p* = 0.043) and MAOA_2 (F(1, 31) = 4.313, *p* = 0.046).

**Figure 6:**
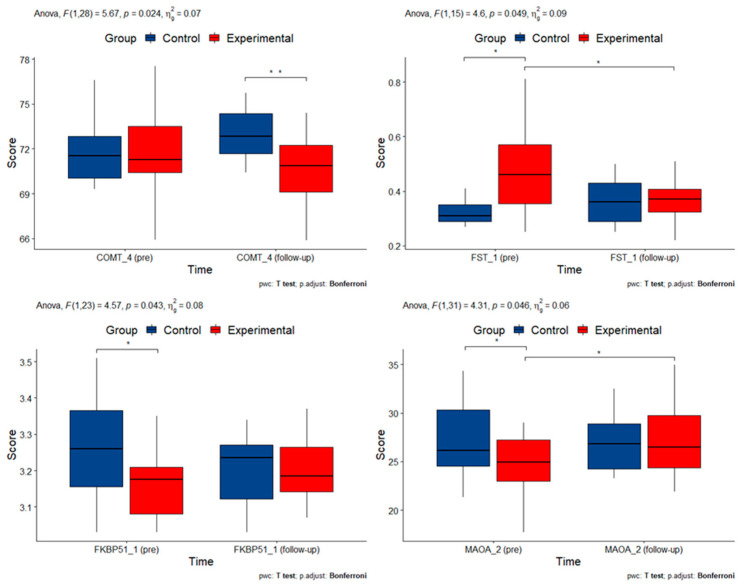
Box plots of measurements of DNA methylation changes with statistically significant interactions. Asterisks denote statistically significant pairwise comparisons.

*p*-values for all candidate genes are summarized in Table 4. Measurements with statistically significant interactions are summarized in Figure 6.

## 4. Discussion

In this study, we investigated the effects of mindfulness-based therapy on clinical presentation and DNA methylation changes in patients with PCOS and high metabolic risk. We expected that patients in the experimental group would have fewer clinical symptoms of PCOS, anxiety, and depression and achieve a higher quality of life after the mindfulness intervention. We also expected that the mindfulness intervention would affect methylation of certain genes associated with PCOS.

PCOS is often associated with a metabolic syndrome defined by central obesity, high triglyceride and low S-HDL levels, hypertension, and inability to control blood glucose. We collected a range of clinical and laboratory data to evaluate the effects of mindfulness intervention on the metabolic syndrome in PCOS. Interestingly, we observed a statistically significant decrease in body weight/BMI and waist circumference. Although there was a statistically significant increase in fasting blood serum glucose level at 0 min in both groups, the change was not clinically meaningful, since all values remained within prediabetes range (5.60 to 6.21 mmol/L). There were no statistically significant differences in MAP, heart rate, cholesterol, S-HDL, or S-LDL. Triglyceride levels, which were significantly higher in the experimental group at baseline, did not differ anymore after the intervention.

Patients who participated in the mindfulness intervention experienced statistically significant improvements in emotional role limitations and measures of general health, as well as significant decreases in measures of pain, as measured by RAND-36. All of these measures of well-being remained stable over time at a six-month follow-up after the intervention. In contrast to the other measures, general health continued to improve, as indicated by the statistically significant difference in this subscale between the end of the intervention and the follow-up.

RAND-36 is a common tool used to assess the effectiveness of mindfulness-based interventions in clinical settings on quality of life [77]. However, results have been mixed. In the study by Morledge and colleagues, mindfulness-based interventions improved physical functioning, general health, social functioning, and emotional role limitations in the normative population as measured by RAND-36 [78]. Van Berkel and colleagues used RAND-36 as a general measure of mental health, and there were no statistically significant effects of a mindfulness-based intervention in the normative population [79]. Mindfulness-based interventions used as adjunctive treatment improved measures of pain, emotional limitations, physical limitations, well-being, energy, and social functioning, but not overall health in addiction patients [80]. Allexandre and colleagues reported statistically significant differences in the normative population in emotional well-being, emotional role functioning, and energy [81]. A mindfulness-based intervention for cancer survivors at metabolic risk reported by Lucas and colleagues showed statistically significant change in RAND-36 subscales for physical well-being but not for psychological well-being [82]. It is likely that mindfulness-based interventions influence psychological and physical well-being in a nonlinear manner.

We expected a statistically significant improvement in the severity of anxious and depressive symptomatology between patients who participated in the MBSR program and the control group that would last six months after the start of therapy. We also hypothesized that the MBSR program would help participants experience and tolerate stress more efficiently. The mindfulness intervention was indeed associated with improvement in anxiety symptomatology as well as subjectively perceived stress, but not in depressive symptomatology. It is possible that the mindfulness intervention provided participants in the experimental group with a new coping mechanism with which to regulate stress, a possibility that is well described in the literature [83,84,85,86]. Another possibility, also supported in the literature, is that mindfulness-based interventions indirectly affect emotion regulation by increasing emotional differentiation [87]. Importantly, this pattern remained stable over time and was still present six months after the intervention. The differential effect of mindfulness intervention on depression and anxiety is surprising, as reviews of MBI efficacy suggest that they alleviate depressive rather than anxiety symptomatology [88].

Interestingly, although there was improvement between the first and second measurement (immediately after therapy) and the first and third measurement (six months after the start of therapy) for anxiety and subjectively perceived stress, as well as for measures of emotional role limitations, pain, and general health, there was no difference between the second measurement after therapy and the six-month follow-up. This suggests that the mindfulness intervention is associated with one-time, long-lasting improvements in psychological well-being, but not with ongoing improvements in mental health.

Our hypothesis was also that six months after the start of therapy, there will be a statistically significant difference in the expression of mindfulness-like traits between patients who underwent the MBSR program and the control group. Indeed, the mindfulness intervention was associated with a statistically significant increase in all mindfulness-like traits, except for non-judging. These improvements remained stable over time but did not increase further.

The results of studies on various cognitive-behavioral and mind–body interventions in women with PCOS suggest that these treatments reduce stress [44,89], anxiety [89,90], depression [89,90,91], quality of life [42,89], eating problems [90], and decrease in body mass index [91], and treatment by a multidisciplinary team is effective in improving healthy lifestyle and achieving long-term weight loss in these women [92]. In addition, when combined with lifestyle modification, these treatments had a greater effect on weight loss and improvement in depression symptoms and quality of life in obese or overweight women with PCOS compared with lifestyle modification alone [93].

Although the effects of mindfulness on adult women with PCOS have not been extensively researched, the results are consistently positive. In a study published in the journal *Stress*, women with PCOS were randomly assigned to participate in an 8-week mindfulness program for stress management that included 30 min of instruction. Twenty-three and fifteen women with PCOS were randomly assigned to the intervention and control groups, respectively. At the end of the study, the women were determined to experience less stress, depression, and anxiety. The women’s salivary cortisol levels also decreased, and life satisfaction and quality of life scores increased only in the intervention group. There was no significant “placebo” effect on outcome measures [89]. Another study examined mindfulness yoga as a popular form of mindfulness intervention. Twenty-two women with PCOS participated in the three-month intervention, 13 in the mindful yoga group and 9 in the control group. The women who participated in the mindful yoga intervention had significantly lower free testosterone levels. In addition, improvements in anxiety and depression were noted, with no weight loss [94].

Regarding the DNA methylation levels of the candidate genes, we expected changes between patients in the experimental and control groups six months after the start of therapy. Candidate genes were selected from two different points of view: Genes already associated with PCOS and its clinical features (CEBPB, EPHX1, EPM2A, FST, IGFBP1, INSR, LHCGR, PPARG1A) and genes involved in the stress response system (SLC6A4, COMT, MAOA, BDNF, NR3C1, FKBP51, TBKBP1) and neurotransmitter signaling (HTR1A, TPH2). We observed a statistically significant effect of mindfulness intervention on three candidate genes selected as stress-associated genes: COMT, MAOA, and FKBP51, and one gene already associated with PCOS, FST. The COMT and MAOA genes encode enzymes that degrade neurotransmitters such as dopamine, serotonin, and norepinephrine, thereby regulating normal brain function. Alterations in COMT and MAOA DNA methylation have previously been linked to stress [95] and various stress-related disorders, such as depression and anxiety [56,96]. Our results on COMT amplicon are similar to those in [95], as stress correlates with increasing DNA methylation. In our study, mindfulness intervention decreased stress and COMT methylation. In the review of stress-related disorders [97] and MAOA methylation, decreased methylation levels were detected in patients compared to controls or in patients before treatment compared to the same patients after treatment. These results are similar to those observed in our study, where MAOA amplicon methylation increased after the mindfulness intervention.

One gene involved in HPA axis function that has been frequently studied in DNA methylation studies is FKBP51. It regulates glucocorticoid activity and response to acute stress through a negative feedback loop in a dynamic process of methylation and demethylation. The protein of FKBP51, FK506-binding protein 5, binds to the glucocorticoid receptor complex, impeding its translocation to the nucleus. In the study of mindfulness-based stress reduction in posttraumatic stress disorder patients, a significant decrease in methylation was observed in patients who responded to the intervention and an increase was observed in patients who did not respond to the intervention. These results suggest that the mediation intervention may affect stress-related molecular pathways at the molecular level [58]. Similarly, a decrease in methylation has been observed in responders and an increase in non-responders of PTSD patients after psychotherapy [63]. In our case, we analyzed similar CpGs as [63], but we observed exactly the opposite pattern of methylation—increase after intervention, while we observed decrease in controls. The only gene selected from the group of PCOS-associated genes that showed a statistically significant change in methylation patterns before and after intervention was FST, follistatin. FST regulates follicular development by binding and neutralizing activins [59], and increased serum levels of FST were detected in PCOS women compared to controls [98]. We attempted to cover the CpG island of FST as much as possible and were able to design three primer pairs. For the primer pair covering the beginning of the island, FST_1, we observed a decrease after the intervention, whereas in the control group, the methylation level remained the same after six months compared with the initial methylation status. On the contrary, in another study with PCOS women, the methylation level of FST showed an increase in methylation at the beginning of the CpG island, but any changes in gene expression could not be detected [59].

## 5. Limitations

Because our sample size was small, the study was exploratory in nature. A series of measurements of DNA methylation of candidate genes was performed, and then the effect of the mindfulness intervention on these genes was estimated. Follow-up studies targeting only the specific measurements that showed significant effects should be conducted to consolidate these results.

## 6. Conclusions

Mindfulness techniques seem promising in ameliorating stress, anxiety, depression, and the quality of life in women with PCOS and could be used as an adjunct method to the conventional management of these women. Drug treatment is crucial for the management of PCOS, but it only targets the symptoms associated with the disease, while the causative treatment for this syndrome is still unknown. Some of these symptoms could also be affected by mindfulness therapy only, which would reduce the number of medications the patient needs to take. It would also be important to determine which aspects of mindfulness therapy are most beneficial in PCOS. With consumer interest in holistic healthcare rising, healthcare providers are required to broaden their knowledge pertaining to the safe and appropriate utilization of these therapies as adjuncts to conventional medical management [42].

## Figures and Tables

**Table 1:** Demographic, clinical, and laboratory characteristics of participants at baseline. Baseline characteristics were compared either using independent samples *t*-test or Mann–Whitney test as appropriate.

Participant Characteristics	Experimental Group	Control Group	*p*-Value
Age (mean (SD))	42.5 y (6.4)	37.7 y (7.4)	0.028
Education (mean (SD))	14.1 y (1.8)	14.1 y (1.6)	0.687
Currently unemployed (n)	3	1	
Metformin treatment (n)	1	1	
Infertility (n)	6	4	
Metabolic syndrome (n)	16	12	
Body weight (mean (SD))	103.8 kg (22.9)	93.0 kg (15.4)	0.084
Body height (mean (SD))	167.8 cm (4.9)	165.0 cm (6.2)	0.105
BMI (mean (SD))	36.2 (7.8)	34.3 (6.3)	0.381
Waist circumference (mean (SD))	115.9 cm (13.4)	108.7 cm (12.2)	0.073
Systolic blood pressure (mean (SD))	126.6 mm Hg (15.4)	123.4 mm Hg (17.4)	0.158
Diastolic blood pressure (mean (SD))	87.1 mm Hg (12.8)	83.3 mm Hg (14.3)	0.364
Heart rate (mean (SD))	77.5 bpm (9.4)	77.8 bpm (10.2)	0.925
Glucose—0 min (mean (SD))	6.2 mmol/L (2.6)	5.6 mmol/L (1.7)	0.279
Glucose—120 min (mean (SD))	7.8 mmol/L (2.5)	7.4 mmol/L (3.5)	0.389
Cholesterol (mean (SD))	5.1 mmol/L (0.9)	5.1 mmol/L (1.1)	0.904
HDL (mean (SD))	1.3 mmol/L (0.3)	1.2 mmol/L (0.2)	0.989
LDL (mean (SD))	3.1 mmol/L (0.9)	3.2 mmol/L (0.9)	0.638
Triglycerides (mean (SD))	1.8 mmol/L (0.8)	1.3 mmol/L (0.6)	0.018
BAI score (mean (SD))	15.3 (12.5)	12.0 (8.8)	0.579
BDI-II score (mean (SD))	10.4 (9.2)	8.9 (9.2)	0.3650
Perceived Stress score (mean (SD))	7.7 (3.1)	7.4 (2.8)	0.756
RAND-36 (mean (SD))			
Physical functioning	74.8 (23.7)	83.1 (17.5)	0.238
Physical role limitations	70.2 (35.0)	76.2 (33.0)	0.598
Emotional role limitations	57.1 (38.2)	77.8 (32.2)	0.067
Energy/Fatigue	50.2 (15.8)	53.3 (14.9)	0.519
Emotional well-being	62.9 (12.0)	61.7 (12.3)	0.762
Social functioning	76.2 (23.7)	79.8 (19.1)	0.786
Pain	65.0 (27.6)	73.6 (22.6)	0.416
General health	53.1 (23.2)	60.7 (17.8)	0.239
FFMQ (mean (SD))			
Observing	3.1 (0.8)	2.8 (0.7)	0.244
Describing (R)	3.0 (1.0)	3.2 (0.9)	0.510
Acting (R)	2.7 (0.9)	2.7 (0.8)	0.886
Non-judging (R)	2.4 (0.8)	2.4 (0.7)	0.961
Non-reactivity	2.8 (0.7)	2.7 (0.6)	0.658

BMI: body mass index; HDL: high density lipoprotein; LDL: low density lipoprotein; BAI: Beck Anxiety Inventory; BDI-II: Beck Depression Inventory-II; RAND-36: RAND-36 Measure of Health-Related Quality of Life questionnaire; FFMQ: Five-Facet Mindfulness Questionnaire; R: reverse score; SD: standard deviation; n: number of participants in the group.

**Table 3:** *p*-values for interactions between group and time for clinical and laboratory measurements. Statistically significant *p*-values are marked in bold.

Participant Characteristics	*p*-Value (ANOVA)
BMI	**0.011**
Waist circumference	**0.024**
Mean arterial blood pressure	0.581
Heart rate	0.631
Glucose—0 min	**0.0460**
Glucose—120 min	**0.039**
Cholesterol	0.811
S-HDL	0.461
S-LDL	0.693
Triglycerides	0.5387

**Table 4:** *p*-values for significant interaction of group and time for methylation of candidate gene amplicons. Table contains only amplicons where the average DNA methylation values could be calculated from available data. Statistically significant *p*-values are marked in bold.

Candidate Gene Amplicon	*p*-Value (ANOVA)
BDNF_95_5	0.797
BDNF_95_2	0.382
BDNF_58_2	0.731
BDNF_58_1	0.845
BDNF_14_1	0.439
BDNF_81_3	0.564
BDNF_81_1	0.795
CEBPB_2	0.696
CEBPB_1	0.557
COMT_2	0.532
COMT_4	**0.024**
COMT_1	0.717
EPHX1_2	0.929
EPM2A_1	0.63
FKBP51_1	**0.043**
FST_1	**0.049**
FST_3	0.491
HTR1A_3	0.115
HTR1A_2	0.684
IGFBP1_1	0.611
IGFBP1_2	0.169
INSR_1	0.91
LHCGR_1	0.952
MAOA_2	**0.046**
MAOA_3	0.862
PPRG1A_1	0.85
SLC6A4_3	0.58
SLC6A4_5	0.403
TPH2_1	0.896

## Data Availability

The data presented in this study are available on reasonable request from the corresponding author. The data are not publicly available due to privacy and ethical restriction.

## Supplementary Materials

The following supporting information can be downloaded at: https://www.mdpi.com/article/10.3390/cimb45040178/s1, Table S1: Amplicon primer sequences and annealing temperatures for candidate genes. Reference [99] is cited in the Supplementary Materials.
