# Supplementary material for: Effects of Mindfulness-Based Therapy on Clinical Symptoms and DNA Methylation in Patients with Polycystic Ovary Syndrome and High Metabolic Risk
Source: Curr Issues Mol Biol. 2023 Mar 24;45(4):2717–37. doi: 10.3390/cimb45040178 (PMC10136994; doi:10.3390/cimb45040178)
Supplement: Supplementary file 1 [file cimb-45-00178-s001.zip › cimb-2271765-supplementary.pdf]

current issues in molecular biology

Supplementary Materials for Article

Effects of Mindfulness-Based Therapy on Clinical Symptoms and DNA Methylation in Patients with Polycystic Ovary Syndrome and High Metabolic Risk

Hana Dema [1], Alja Videtič Paska [2,*], Katarina Kouter [2], Mojca Katrašnik [2], Mojca Jensterle [3,4], Andrej Janež [3,4], Aleš Oblak [5], Borut Škodlar [5,6] and Jurij Bon [5,6,*]

[1] Department of Health Care Quality, University Medical Centre Ljubljana, SI-1000 Ljubljana, Slovenia
[2] Institute for Biochemistry and Molecular Genetics, Faculty of Medicine, University of Ljubljana, SI-1000 Ljubljana, Slovenia
[3] Department of Endocrinology, Diabetes and Metabolic Diseases, University Medical Centre Ljubljana, SI-1000 Ljubljana, Slovenia
[4] Department of Internal Medicine, Faculty of Medicine, University of Ljubljana, SI-1000 Ljubljana, Slovenia
[5] University Psychiatric Clinic Ljubljana, SI-1260 Ljubljana, Slovenia
[6] Department of Psychiatry, Faculty of Medicine, University of Ljubljana, SI-1000 Ljubljana, Slovenia
* Correspondence: alja.videtic@mf.uni-lj.si (A.V.P.); jurij.bon@mf.uni-lj.si (J.B.)

Citation: Dema, H.; Videtič Paska, A.; Kouter, K.; Katrašnik, M.; Jensterle, M.; Janež, A.; Oblak, A.; Škodlar, B.; Bon, J. Effects of Mindfulness-Based Therapy on Clinical Symptoms and DNA Methylation in Patients with Polycystic Ovary Syndrome and High Metabolic Risk. *Curr. Issues Mol. Biol.* **2023**, *45*, 2717–2737. https://doi.org/10.3390/cimb45040178

Academic Editors: Hsien-Ming Wu

Received: 23 February 2023
Revised: 19 March 2023
Accepted: 23 March 2023
Published: 24 March 2023

Copyright: © 2023 by the authors. Submitted for possible open access publication under the terms and conditions of the Creative Commons Attribution (CC BY) license (https://creativecommons.org/licenses/by/4.0/).

Abstract: Polycystic ovary syndrome (PCOS) is an endocrine and metabolic disorder affecting women of reproductive age. Research has shown that epigenetic alterations such as DNA methylation may play a role in the development and progression of abnormal ovarian function and metabolic disorders in PCOS. Among other epigenetic changes, studies have identified specific genes (related with insulin signalling and steroid hormone metabolism) that are methylated in women with PCOS. DNA methylation appears to respond to various interventions aimed at altering health and lifestyle factors. We tested the efficacy of a mindfulness-based stress reduction program (MBSR) in PCOS patients. We examined its effects on anthropometric measurements, mental health and wellbeing, and alterations in DNA methylation in peripheral blood. MBSR was associated with a reduction in body mass index, waist circumference and blood glucose level, an improvement in subjectively perceived general health, emotional role limitation, and levels of pain, as well as mindfulness-like traits. MBSR reduced the expression of anxious symptomatology and subjectively perceived stress. Methylation changes were observed in four genes: COMT, FST, FKBP51, and MAOA. We conclude that MBSR may be a useful supplementary therapy to mitigate the deleterious effects of PCOS on mental health.

Keywords: mindfulness-based stress reduction program, polycystic ovary syndrome, epigenetics, DNA methylation, candidate genes, depression, anxiety

Table S1. Amplicon primer sequences and annealing temperatures for candidate genes. Illumina universal sequences are presented in grey shade and are followed by the sequence-specific primers that align on the target bisulfite converted DNA sequence.

Amplicon	Forward or reverse primer	Primer Sequence	Annealing temperature / °C
SLC6A4_3	Forward primer	**TCGTCGGCAGCGTCAGATGTGTATAAGAGACAG**AGTTAAAGAGTAGGAAAGTTAGGATTTT	54.0
	Reverse primer	**GTCTCGTGGGCTCGGAGATGTGTATAAGAGACAG**ACCCTCACATAATCTAATCTCTAAA	
SLC6A4_5	Forward primer	**TCGTCGGCAGCGTCAGATGTGTATAAGAGACAG**TTTGAGTAGTTGGGAATATAAG	52.0
	Reverse primer	**GTCTCGTGGGCTCGGAGATGTGTATAAGAGACAG**AAAACCCTTATCTAATTCCTCTC	
COMT_1[a]	Forward primer	**TCGTCGGCAGCGTCAGATGTGTATAAGAGACAG**GTTTTTAATTTTGTATAGGTAAGAT	54.0
	Reverse primer	**GTCTCGTGGGCTCGGAGATGTGTATAAGAGACAG**TACCCTCCCTACCCACAAC	
COMT_2[a]	Forward primer	**TCGTCGGCAGCGTCAGATGTGTATAAGAGACAG**GGTTATTTGTGGTTAGAAGTAGTT	54.0
	Reverse primer	**GTCTCGTGGGCTCGGAGATGTGTATAAGAGACAG**ACTACCCCAAAAACCCCAC	
COMT_4[a]	Forward primer	**TCGTCGGCAGCGTCAGATGTGTATAAGAGACAGNN**TGTAGGAGGAGTATAGAGTATTGG	62.0
	Reverse primer	**GTCTCGTGGGCTCGGAGATGTGTATAAGAGACAGNN**TCATAACCCACTCCTTCTACT	
BDNF_81_1[a]	Forward primer	**TCGTCGGCAGCGTCAGATGTGTATAAGAGACAG**TGAGGGTAGGTAAAGGGTAGT	55.3
	Reverse primer	**GTCTCGTGGGCTCGGAGATGTGTATAAGAGACA**ACTCTCCCAAAAAACCCTAC	
BDNF_81_2[a]	Forward primer	**TCGTCGGCAGCGTCAGATGTGTATAAGAGACAG**TTATATAGGTTTTTGTGGGTAATTAG	55.0
	Reverse primer	**GTCTCGTGGGCTCGGAGATGTGTATAAGAGACAG**AAAAAAAAAAAACTTCTTAAAAAAAT	
BDNF_81_3[a]	Forward primer	**TCGTCGGCAGCGTCAGATGTGTATAAGAGACAG**TTTTTAGTTATGATGGGGGAGG	58.0
	Reverse primer	**GTCTCGTGGGCTCGGAGATGTGTATAAGAGACAG**CAAATCACACCTAAAACTCC	
BDNF_14_1[a]	Forward primer	**TCGTCGGCAGCGTCAGATGTGTATAAGAGACAG**GAGTTTATTTAGTATTTTGGATAGA	55.3
	Reverse primer	**GTCTCGTGGGCTCGGAGATGTGTATAAGAGACAG**AAAAATCTATTCCAACCTACACC	
BDNF_58_1[a]	Forward primer	**TCGTCGGCAGCGTCAGATGTGTATAAGAGACAGTTTTTTTAAGGGAAGGGGAGTT**	54.0
	Reverse primer	**GTCTCGTGGGCTCGGAGATGTGTATAAGAGACAGAAACTAAAAATATTCTTCTCCACC**	
BDNF_58_2[a]	Forward primer	**TCGTCGGCAGCGTCAGATGTGTATAAGAGACAGATAGAGTTATTAATTAGTTGGA**	55.0

	Reverse primer	**GTCTCGTGGGCTCGGAGATGTGTATAAGAGACAG-TAAATCCCTAAACTCCCTAAAA**	
BDNF_95_1[a]	Forward primer	**TCGTCGGCAGCGTCAGATGTGTATAAGAGA-CAGGGTTTTAATGAGATATTTAT**	58.0
	Reverse primer	**GTCTCGTGGGCTCGGAGATGTGTATAAGAGA-CAGAAAATCCCCCAATCAACTCTCT**	
BDNF_95_2[a]	Forward primer	**TCGTCGGCAGCGTCAGATGTGTATAAGAGACAG**AAATGTT-GTTATTATTTTGATTGAATT	54.0
	Reverse primer	**GTCTCGTGGGCTCGGAGATGTGTATAAGAGA-CAG**AACACCCAAATTCTCTAAAAAA	
BDNF_95_5[a]	Forward primer	**TCGTCGGCAGCGTCAGATGTGTATAAGAGA-CAGNN**TTTTTTAGAGAATTTGGGTGT	56.7
	Reverse primer	**GTCTCGTGGGCTCGGAGATGTGTATAAGAGA-CAGNN**AAACCTATCCTCACCTCCT	
TPH2_1[b]	Forward primer	**TCGTCGGCAGCGTCAGATGTGTATAAGAGACAG**AGGTTT-GTAATTTGATTGTGGT	52.0
	Reverse primer	**GTCTCGTGGGCTCGGAGATGTGTATAAGAGACAG**CCAA-TATTTACTAAAAAACATCATCA	
HTR1A_1[b]	Forward primer	**TCGTCGGCAGCGTCAGATGTGTATAAGAGACAG**TTTTAGA-TATTTTTGGGATTGGAGAT	55.0
	Reverse primer	**GTCTCGTGGGCTCGGAGATGTGTATAAGAGACAG-**CAAAAACCCAAACAAAAAATTCTTAC	
HTR1A_2[b]	Forward primer	**TCGTCGGCAGCGTCAGATGTGTATAAGAGACAG-**TGGTTTATTGGTTTTTTTATTTTTAT	50.0
	Reverse primer	**GTCTCGTGGGCTCGGAGATGTGTATAAGAGA-CAG**CTCCATTCACACTCTTCTTAAAC	
MAOA_1[b]	Forward primer	**TCGTCGGCAGCGTCAGATGTGTATAAGAGACAG**GA-TAGTTTTTAGTTAAAATTAAAGAATGAA	50.0
	Reverse primer	**GTCTCGTGGGCTCGGAGATGTGTATAAGAGACAG-**TACCCACTCTTAAAAACCAACC	
MAOA_2[b]	Forward primer	**TCGTCGGCAGCGTCAGATGTGTATAAGAGACAG**GGGAGTT-GATAGAAGGGTTTTTT	59.0
	Reverse primer	**GTCTCGTGGGCTCGGAGATGTGTATAAGAGACAG**CAAAC-CATAACTACACTACACTCTC	
CEBPB_1	Forward primer	**TCGTCGGCAGCGTCAGATGTGTATAAGAGACAG**TGGTTTGG-GATTTAGTATGTT	52.1
	Reverse primer	**GTCTCGTGGGCTCGGAGATGTGTATAAGAGACAG**TTCTTAC AATTCTTACCCCC	
CEBPB_2	Forward primer	**TCGTCGGCAGCGTCAGATGTGTATAAGAGACAG**GATTGGG AAGGGGATTTAT	50.2
	Reverse primer	**GTCTCGTGGGCTCGGAGATGTGTATAAGAGACAG**ACCACT TCCATAAATTTAAAAACA	
EPHX1_2	Forward primer	**TCGTCGGCAGCGTCAGATGTGTATAAGAGACAG**TGTAGAG GGATTTGTAGTTGGTT	55.3
	Reverse primer	**GTCTCGTGGGCTCGGAGATGTGTATAAGAGACAG**ACTTAA ATAACAAATTAAATACATTTC	

EPM2A_1	Forward primer	**TCGTCGGCAGCGTCAGATGTGTATAAGAGACAG**TTGGGGT GGTGGTGTTAT	55.3
	Reverse primer	**GTCTCGTGGGCTCGGAGATGTGTATAAGAGACAG**ATACCT TCCCAAAAAAACTCTCC	
FKBP51_F1[c]	Forward primer	**TCGTCGGCAGCGTCAGATGTGTATAAGAGACAG**AGGGGGT GTTAGTTTTTATTATTTTTT	50.2
	Reverse primer	**GTCTCGTGGGCTCGGAGATGTGTATAAGAGACAG**ACTCCG CTAACCCTTCAAC	
FST_1	Forward primer	**TCGTCGGCAGCGTCAGATGTGTATAAGAGACAG**AGGATAG AAATTGGGGAGTTTT	55.3
	Reverse primer	**GTCTCGTGGGCTCGGAGATGTGTATAAGAGACAG**TACCTA ACTTTACAACCACCCC	
FST_2	Forward primer	**TCGTCGGCAGCGTCAGATGTGTATAAGAGACAG**TTTGAAGT GGGTGTTTTTTTT	50.2
	Reverse primer	**GTCTCGTGGGCTCGGAGATGTGTATAAGAGA-CAG**ATCAAATAACATTTCCCACCTT	
FST_3	Forward primer	**TCGTCGGCAGCGTCAGATGTGTATAAGAGACAG**AGAAGGT GGGAAATGTTATTTG	55.3
	Reverse primer	**GTCTCGTGGGCTCGGAGATGTGTATAAGAGA-CAG**AACAACAAAAAACAAAACCCAC	
IGFBP1_1	Forward primer	**TCGTCGGCAGCGTCAGATGTGTATAAGAGACAG**GGTTT-GTGTTTTTTATAAGGTG	55.3
	Reverse primer	**GTCTCGTGGGCTCGGAGATGTGTATAAGAGA-CAG**ATCAACAAAAACAATACCAACCA	
IGFBP1_2	Forward primer	**TCGTCGGCAGCGTCAGATGTGTATAAGAGACAG**GGTTGG-TATTGTTTTTGTTGA	50.2
	Reverse primer	**GTCTCGTGGGCTCGGAGATGTGTATAAGAGACAG-**CAAAAATTACTACTCCCCC	
INSR_1	Forward primer	**TCGTCGGCAGCGTCAGATGTGTATAAGAGACAG**GATGGG-GAGAGGATTTTATTTA	50.2
	Reverse primer	**GTCTCGTGGGCTCGGAGATGTGTATAAGAGA-CAG**AACCCAATAACCCCACTTC	
LHCGR_1	Forward primer	**TCGTCGGCAGCGTCAGATGTGTATAAGAGACAG**AGTAG-GAAGGAGGTTATTGGGTTAT	56.7
	Reverse primer	**GTCTCGTGGGCTCGGAGATGTGTATAAGAGACAG**C-TACAACAACAACAACAACTTCAAC	
NR3C1_1[c]	Forward primer	**TCGTCGGCAGCGTCAGATGTGTATAAGAGACAG**TTTTT-GAAGTTTTTTTAGAGGG	55.3
	Reverse primer	**GTCTCGTGGGCTCGGAGATGTGTATAAGAGA-CAG**AATTTCTCCAATTTCTTTTCTC	
PPRG1A_1	Forward primer	**TCGTCGGCAGCGTCAGATGTGTATAAGAGACAG**TAG-GATTGTGTGTGGAGTTGTT	52.1
	Reverse primer	**GTCTCGTGGGCTCGGAGATGTGTATAAGAGACAG**CCAAC-TACCCAAAAAAAACACTC	
TBKBP1_1	Forward primer	**TCGTCGGCAGCGTCAGATGTGTATAAGAGACAG-**GAAGGTATTTATTGATTGA	52.1

Reverse primer	**GTCTCGTGGGCTCGGAGATGTGTATAAGAGA-CAG**AATTCCCAAAACTATCTC

[a]Primer design according to Kouter et al. [99].
[b]Primer design according to Kouter et al. [60].
[c]Primer design according to Yehuda et al. [63].

60. Kouter, K.; Zupanc, T.; Videtič Paska, A. Targeted sequencing approach: Comprehensive analysis of DNA methylation and gene expression across blood and brain regions in suicide victims. *World J Biol Psychiatry* **2023**, *24*, 12-23. 10.1080/15622975.2022.2046291

63. Yehuda, R.; Daskalakis, N.P.; Desarnaud, F.; Makotkine, I.; Lehrner, A.L.; Koch, E.; Flory, J.D.; Buxbaum, J.D.; Meaney, M.J.; Bierer, L.M. Epigenetic biomarkers as predictors and correlates of symptom improvement following psychotherapy in combat veterans with ptsd. *Front Psychiatry* **2013**, *4*, 118. 10.3389/fpsyt.2013.00118

99. Katarina Kouter, M.N.P., Gordana Nedic Erjavec, Tina Milos, Lucija Tudor,; Suzana Uzun, N.M., Nela Pivac, Alja Videtic Paska. Difference in methylation and expression of brain-derived neurotrophic factor in alzheimer’s disease and mild cognitive impairment. *Biomedicines 2023* **2023**, *11*, 235. 10.3390/biomedicines11020235
